# Recent Advances in Stimuli‐Responsive Materials and Soft Robotic Actuators for Bioelectronic Medicine

**DOI:** 10.1002/adma.202417325

**Published:** 2025-04-15

**Authors:** Chaoqun Dong, George G. Malliaras

**Affiliations:** ^1^ Electrical Engineering Division Department of Engineering University of Cambridge Cambridge CB3 0FA United Kingdom

**Keywords:** bioelectronics, neural implants, soft robotic bioelectronics, soft actuators, stimuli‐responsive materials

## Abstract

Bioelectronic medicine uses implantable electronic devices to interface with electrically active tissues and transform the way disease is diagnosed and treated. One of the biggest challenges is the development of minimally invasive devices that can be deployed to patients at scale. Responsive materials and soft robotic actuators offer unique opportunities to make bioelectronic devices with shape actuation, promising to address the limitations of existing rigid and passive systems, including difficult deployment, mechanical mismatch with soft tissues, and limited adaptability in minimally invasive settings. In this review, an overview is provided of smart materials and soft robotic technologies that show promises for implantable use, discussing advantages and limitations of underlying actuation mechanisms. Examples are then presented where soft actuating mechanisms are combined with microelectrodes to create shape actuating bioelectronic devices. Opportunities and challenges for next‐generation intelligent bioelectronic devices assisted by responsive materials and soft robotic actuators are then discussed. These innovations may allow electronic implants to safely navigate to target areas inside the body and establish large area and spatiotemporally controlled interfaces for diagnostic or therapeutic procedures that are minimally invasive.

## Graphical abstract

This review explores the integration of responsive materials and soft robotic actuators with implantable electronics to address key challenges in bioelectronic medicine. By enabling shape actuation, these technologies improve deployment, adaptability, and accuracy in minimally invasive procedures. The review discusses actuation mechanisms, device designs, and future opportunities for intelligent, responsive implants with enhanced therapeutic and diagnostic capabilities.

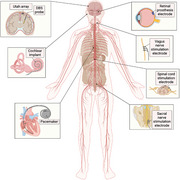

## 1 Introduction

**Figure 1:**
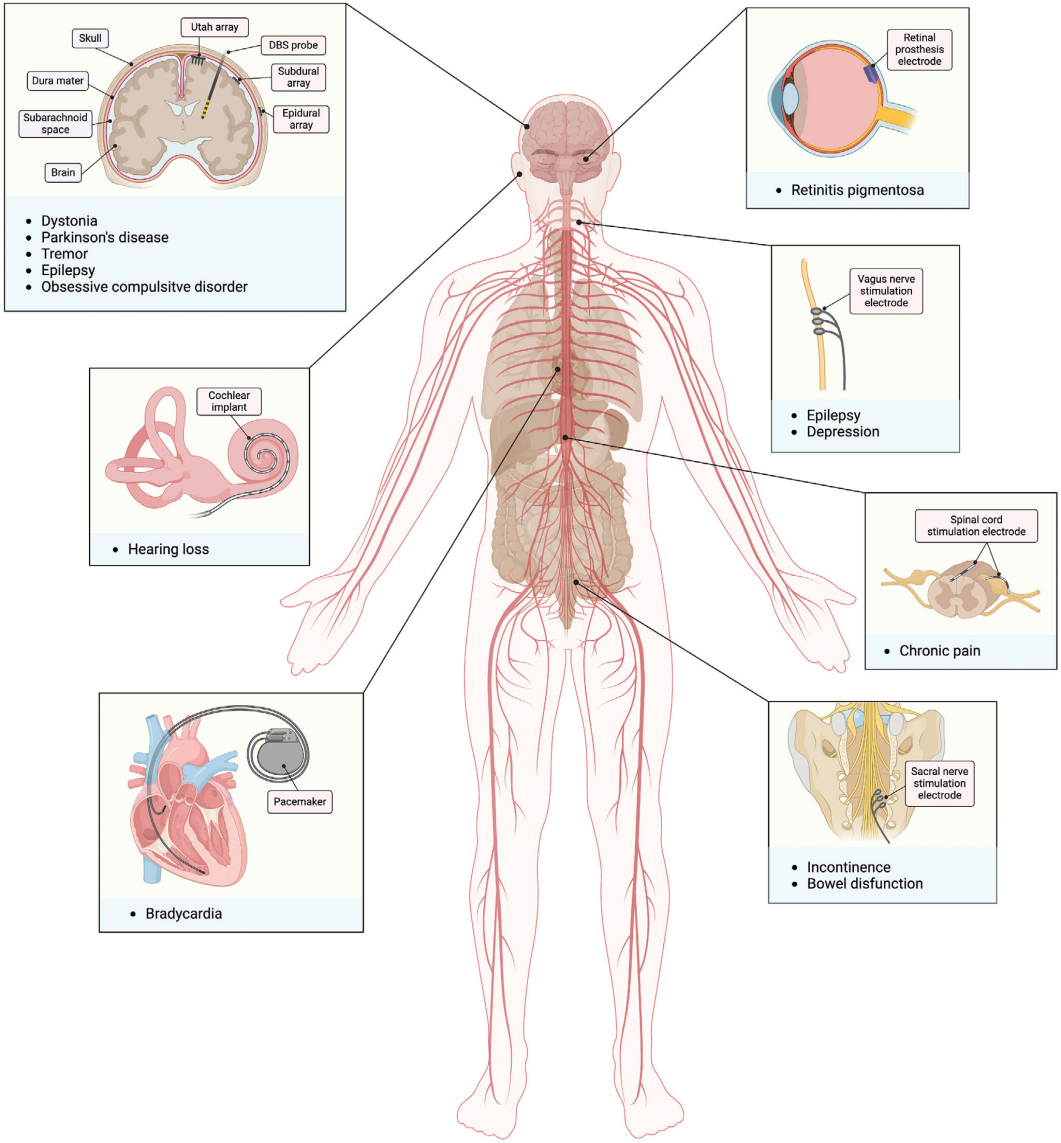
Schematic representation of different implants for bioelectronic medicine applications.

Advances in the precise detection and modulation of electrical signals in biological tissues have catalyzed bioelectronic medicine as a promising therapeutic approach.^[^ ^1^, ^2^, ^3^ ^]^ Unlike conventional drug therapies, bioelectronic medicine utilizes implantable devices to deliver electrical current pulses to specific biological pathways in the brain,^[^ ^4^, ^5^, ^6^ ^]^ spinal cord,^[^ ^7^, ^8^ ^]^ peripheral nerves,^[^ ^9^, ^10^ ^]^ and muscles.^[^ ^11^, ^12^ ^]^ These devices have demonstrated success in treating conditions ranging from hearing loss^[^ ^13^ ^]^ to epilepsy,^[^ ^14^ ^]^ Parkinson's disease,^[^ ^15^, ^16^, ^17^ ^]^ and bladder dysfunction,^[^ ^18^, ^19^ ^]^ by stimulating neural circuits involved in these conditions (**Figure** **1**).^[^ ^20^ ^]^ By integrating medicine, neuroscience and engineering, bioelectronic medicine offers the potential to revolutionize the management of an increasing number of chronic and life‐threatening diseases.

Existing implantable devices provide impressive durability and ease of surgical handling.^[^ ^21^ ^]^ However, their bulkiness, mechanical stiffness and planar design impede seamless integration with the soft, curved surfaces of biological tissues. This misalignment compromises both the resolution and stability required for long‐term recordings of neural signals.^[^ ^22^ ^]^ Recent innovations in mechanically flexible, polymer‐based bioelectronics offer promising solutions to minimize mechanical mismatch at the tissue/implant interface, reduce immune reactions, and enhance the precision of bidirectional interaction.^[^ ^23^, ^24^, ^25^ ^]^ One of the main challenges of using these flexible devices is the difficulty in handling and surgical implantation. Soft devices lack the rigidity necessary for direct implantation into the brain and often require stiff shuttle devices to assist insertion,^[^ ^26^, ^27^ ^]^ which may increase trauma and risk of displacement or damage to the implant during shuttle withdrawal. The invasiveness is also accompanied by complex implantation procedures, such as using metallic surgical tools to place small, 2D planar nerve cuff electrodes onto delicate, 3D nerves, which could prolong surgery time and increase the risk of irreversible nerve damage.^[^ ^28^ ^]^ These exampled challenges highlight the need for innovative device designs that facilitate gentle implantation while accommodating the complex geometries of biological tissues. The clinical demand for minimally invasive surgery has been rapidly increasing, as it improves both the effectiveness of treatments and the overall experience for both surgeons and patients.^[^ ^29^ ^]^ These procedures aim for smaller incisions, along with safer, more efficient, and faster operations. However, current implantable electrodes are often large, requiring incisions larger than the devices themselves, which elevates the risk of infection, pain, and prolonged recovery.^[^ ^30^ ^]^ While smaller devices could reduce invasiveness, they often sacrifice electrode count and coverage, potentially missing valuable recordings from surrounding neurons and resulting in less complete or precise representations of neural activity. This limitation may also impair therapeutic outcomes in treatments that rely on stimulating broader or more distributed neural circuits, as seen in applications for epilepsy. Thus, balancing device miniaturization with sufficient electrode coverage remains a key challenge in developing minimally invasive neural implants. There is a critical need to develop novel devices that require less invasive surgery to implant, for example, implantation through a burr hole instead of a craniotomy, and that can expand or navigate to target sites without compromising the quality of the tissue/electrode interface.^[^ ^31^ ^]^

**Figure 2:**
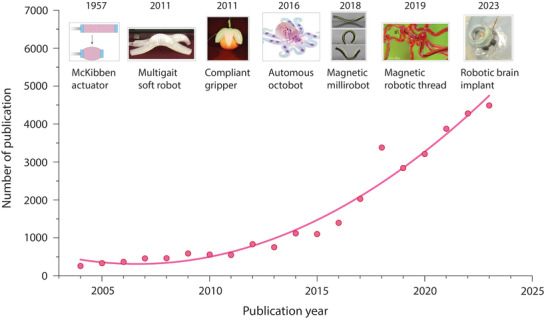
Timeline of soft robotics development. The plot shows the number of publications reporting soft robotics during the past two decades. Data was collected from Web of Science with the following search commands: soft AND (robot OR robots OR robotics OR actuator OR actuators OR actuation). The images show several examples of soft actuators. Image for “Multigait soft robot”^32^: Reproduced with permission. Copyright (2011) National Academy of Sciences. Image for “Compliant gripper”^33^: Reproduced with permission. Copyright 2011 WILEY‐VCH Verlag GmbH & Co. KGaA, Weinheim. Image for “Automous octobot”: Reproduced with permission.^[^ ^34^ ^]^ Copyright 2016, Springer Nature. Image for “Magnetic millirobot”: Reproduced with permission.^[^ ^35^ ^]^ Copyright 2018, Springer Nature. Image for “Magnetic robotic thread”: Reproduced with permission.^[^ ^36^ ^]^ Copyright 2019, The American Association for the Advancement of Science. Image for “Robotic brain implant”.^[^ ^37^ ^]^ Copyright 2023, The American Association for the Advancement of Science.

In nature, many biological systems exhibit remarkable adaptive capacities that allow them to sense environmental changes and respond effectively. Examples include the octopus squeezing through small spaces, the unfolding of pinecones during drying for seed dispersal, the rapid snapping of the Venus flytrap to capture insects, and the chiral growth of tendrils in climbing plants to optimize sunlight exposure. Similar actuation has been replicated in human‐made devices. Scientists and engineers have developed soft robotic systems that mimic nature's behaviors by utilizing stimuli‐responsive materials and actuation mechanisms. Unlike rigid counterparts, these soft systems can undergo significant changes in shape, volume, and stiffness.^[^ ^34^, ^38^, ^39^ ^]^ The history of soft robotics dates back to 1957, with the development of pneumatically driven McKibben actuators for artificial limbs.^[^ ^40^ ^]^ The field experienced a significant breakthrough following the demonstration of a multigait soft robot by Whitesides and colleagues,^[^ ^32^ ^]^ constructed using elastic materials (**Figure** **2**). Since then, numerous soft actuation mechanisms have been developed, allowing devices to perform complex tasks that were previously unachievable with conventional rigid actuators, such as grasping fragile objects or navigating delicate blood vessels without causing damage.^[^ ^32^, ^33^, ^34^, ^35^, ^36^, ^37^ ^]^ Currently, soft actuators can conform more naturally to biological tissues, offer enhanced comfort and interaction when integrated into wearable devices.^[^ ^41^, ^42^, ^43^ ^]^ Additionally, some highly integrated soft robotic systems can provide real‐time feedback, enabling the detection of internal strain and the sensation of external surface contours and softness, which facilitates closed‐loop control for precise actuation.^[^ ^44^ ^]^ Yet, the application of soft actuators and smart materials in implantable devices remains underexplored and faces some critical challenges.^[^ ^45^, ^46^ ^]^ Although the milestones shown in Figure 2 may not all depict direct bioelectronic applications, they represent various actuation strategies that can be or have already been, integrated into implantable bioelectronics, thereby highlighting the synergy between soft robotics and bioelectronic medicine. For instance, multigait soft robots and compliant grippers have inspired new strategies for minimally invasive procedures, while magnetic actuators hold promises to be integrated with microelectronics for wireless control.

Given the very early‐stage nature of this field and its interdisciplinary requirements, advancing soft intelligent bioelectronics will necessitate collaboration across a wide range of disciplines, ranging from surgery, neuroscience, and biomedical science to materials science, robotics, electrical engineering, and other engineering fields. This Review aims to bridge the gaps between these disciplines by summarizing recent progress in integrating responsive materials and soft actuators with thin‐film electronics to overcome the limitations of passive and rigid implants. These devices can dynamically adjust their mechanical properties or form factors in response to stimuli such as body temperature, biofluids, or externally applied magnetic or electric fields. This adaptability contributes to establishing minimally invasive, high‐quality interfaces between bioelectronic devices and biological tissues. In the following sections, we will introduce some stimuli‐responsive materials and actuators with potential for implantable applications, followed by a discussion of several state‐of‐the‐art studies that combine shape‐morphing devices and soft robotic actuators with bioelectronics. Finally, we will envision potential features of next‐generation intelligent bioelectronics and highlight the key challenges that must be addressed to advance the field further.

## 2 Promising Soft Actuation Strategies for Implantable Use

Materials selection and structural design are critical in constructing implantable soft actuators that convert various energy inputs into mechanical outputs. In this section, we introduce soft actuation mechanisms that hold potential for implantable use, which can be broadly categorized into two types. The first type involves smart materials that respond to physiological stimuli, such as biofluids, body temperature, or pH. These materials require no external control for the function, making the devices simpler in structure and easier to fabricate. The second type relies on external control and energy input, which offers greater complexity but provides significant advantages in programmability, controllability, and repeatability, making them great candidates to perform complex tasks or to reach hard‐to‐access sites within the body. In **Table** **1**, we summarize the critical features of different actuation strategies, along with their biocompatibility and safety considerations for biomedical use.

**Table 1:** Comparison of actuation mechanisms.

Actuation Mechanism	Operating Principle	Device Size	Force Output	Strain	Actuation Speed/Response Time	Voltage/Power Requirement	In Vivo or In Vitro	Biocompatibility & Safety	Refs.
**Pneumatic/hydraulic**	Pressurized fluid within elastomeric chambers	mm–cm	up to 0.5–2 N, design‐dependent	≈10–100%	≈0.05–1 s	External pneumatic source (0.05–0.4 MPa)	In vitro and in vivo	Elastomeric materials generally biocompatible; risk of tissue damage if over‐pressurized or poorly controlled	[32, 60, 61, 62]
**Tendon‐driven**	Pulling on embedded cables (motor‐driven or manual)	mm–cm	up to 1–10 N, depending on cable, motor, and lever arm	≈10–30%	≈0.1–2 s (motor speed and geometry dependent)	Low voltage DC motor or manual pull	In vivo (some surgical tools)	Generally safe if using biocompatible cable materials; friction & wear at cable tissue interface must be minimized	[63, 64, 65]
**Dielectric Elastomer Actuators**	Electric field compresses elastomeric structure	µm–cm	Up to 1 N	>100%	≈0.001–0.1 s	0.5‐20 kV	In vitro	Requires low‐permeability encapsulation; high voltage can cause local heating or dielectric breakdown	[66, 67, 68]
**Ionic electroactive polymer actuators**	Ion migration changes volume of polymer film	µm–cm	≈mN	3–30%	0.1–10 s	0.5‐5 V	In vitro and in vivo	Generally biocompatible polymers; force output is generally low	[69, 70, 71]
**Light‐driven**	Photothermal or photochemical effects	mm–cm	≈mN	Up to ≈30–80%	From <1 s to minutes (wavelength and intensity dependent)	External light source	Mostly in vitro	Limited tissue penetration (especially UV‐visible); NIR can reach a few cm; must avoid thermal damage	[72, 73, 74]
**Acoustic/ultrasonic**	Ultrasound waves generate movement, vibration, or deformation	µm–mm	mN–N	<5–10%	≈1–2 s (to achieve significant displacement)	External ultrasound	In vitro and in vivo	Possible localized heating if intensity too high; deeper penetration than visible light	[75, 76, 77]
**Shape memory polymers**	Thermally triggered phase transition	µm–cm	0.1–1^[^ ^78^ ^]^ N	Up to 50–200%	From 10 s to minutes	≈37–60 °C activation (body temperature or external heating)	In vitro and in vivo	Biocompatible polymers available; risk of overheating tissues if poorly regulated	[78, 79, 80, 81]
**Swelling‐based hydrogels**	Polymer network absorbs fluid, increasing volume	µm–cm	Low force	Up to 100–1000%	from seconds to hours	None (if triggered by environment change)	In vitro and in vivo	Generally good biocompatibility; over swelling if not well‐controlled	[82, 83, 84]
**Magnetic**	Embedded magnetic particles activated by external magnetic field	µm–cm	mN to N	≈5–50%	<1 s (depending on field and coil design)	External magnets	In vitro and in vivo	Generally safe for low‐intensity fields; potential interference with MRI	[85, 86]

### 2.1 Intelligent Materials Responsive to Physiological Conditions

Stimuli‐responsive materials, also referred to as smart or intelligent materials, change their physical or chemical properties when exposed to external stimuli. These predictable changes—such as alterations in shape, size, or mechanical properties—make them particularly valuable for biomedical devices requiring adaptive behavior.

#### 2.1.1 Water‐Responsive Materials

**Figure 3:**
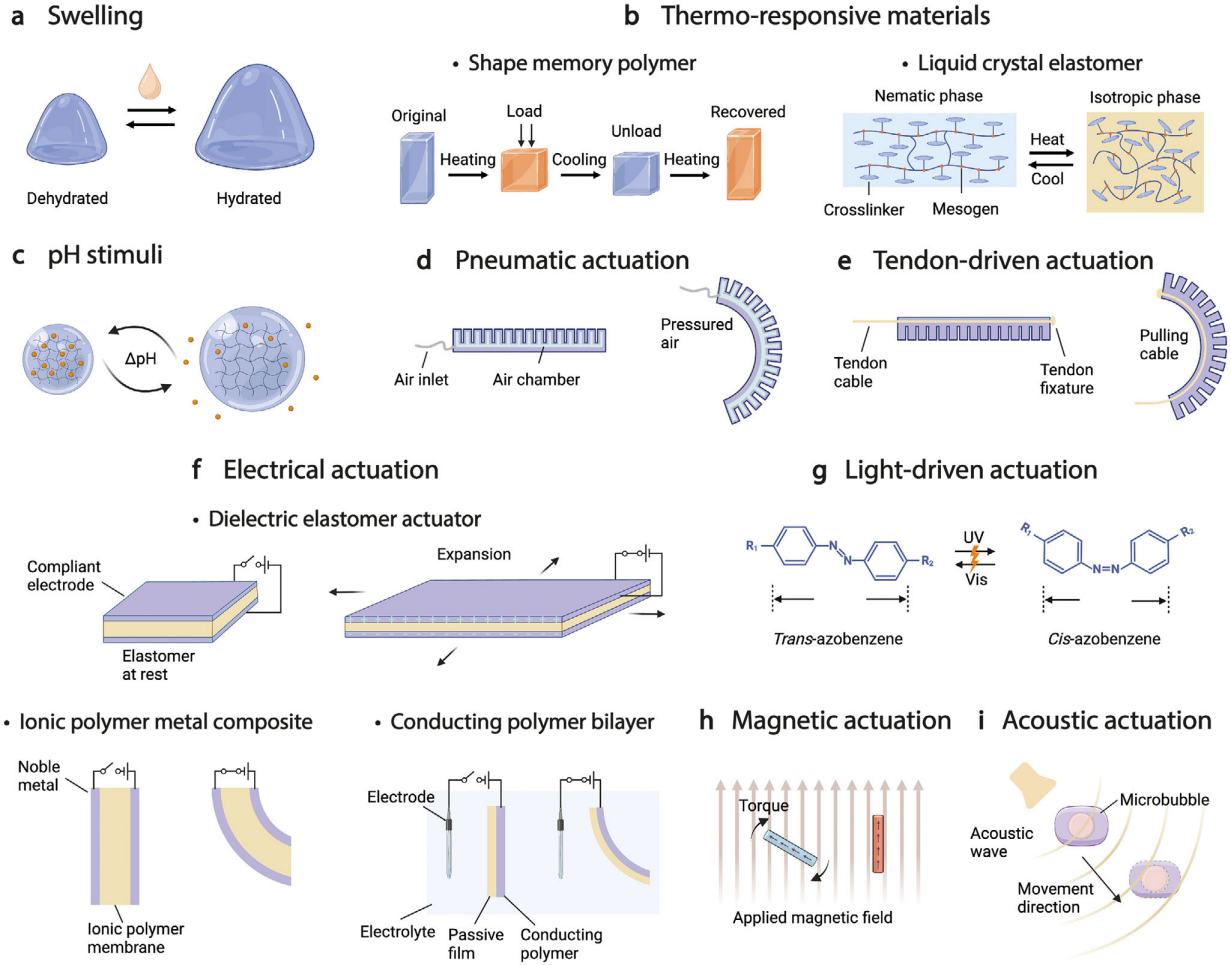
Working mechanisms of soft actuation that are promising for implantable use. a–c) Intelligent materials responsive to physiological conditions: (a) swelling in biofluid, (b) shape morphing triggered by body temperature, and (c) pH responsive materials. d–h) Reversible soft actuation in body environment: (d) pneumatic, (e) tendon‐driven, (f) electrical, (g) light‐driven, (h) magnetic, and (i) acoustic.

Swelling can adjust the mechanical properties of polymers by absorbing water from biofluids like blood or cerebrospinal fluid (**Figure** **3**a).^[^ ^47^, ^48^ ^]^ Hydrophilic polymer networks, such as alginate, agarose, chitosan, hyaluronic acid, poly(vinyl alcohol) (PVA), poly(ethylene glycol) (PEG), and cellulose, facilitate this process by retaining water through hydrogen bonds and electrostatic interactions.^[^ ^49^, ^50^, ^51^ ^]^ The swelling can be used to modulate stiffness, exert force, or induce shape changes like bending or elongation.^[^ ^52^ ^]^ The rate and extent of swelling, along with the resulting behaviors, can be finely controlled by adjusting the material's composition, crosslinking, and structure. Hydrogels typically swell and shrink uniformly in all directions; however, achieving directional bending or folding often requires inhomogeneous deformation. Constructing bilayer or trilayer structures is a common strategy to introduce such inhomogeneity for directed bending or folding of hydrogel films.^[^ ^53^ ^]^ Additionally, methods like selective patterning enable more complex shape deformations.^[^ ^54^ ^]^ The biocompatibility and autonomous response without the need for external equipment make these materials ideal for applications like drug delivery,^[^ ^55^, ^56^ ^]^ tissue scaffolding,^[^ ^57^ ^]^ neural probes,^[^ ^58^, ^59^ ^]^ and other implants requiring mechanical adaptability or localized force in physiological conditions.

#### 2.1.2 Thermo‐Responsive Materials

Poly(N‐isopropylacrylamide) (PNIPAM) is among the most extensively studied thermo‐responsive polymers. Its temperature‐dependent behavior stems from the interplay between the isopropyl group's hydrophobicity and the amide group's hydrophilicity, both of which vary with external temperature.^[^ ^87^ ^]^ When heated above its lower critical solution temperature (LCST, ∼32 °C), the hydrophobic effect of the isopropyl group prevails, reducing intermolecular distances and causing the polymer to de‐swell and shrink.^[^ ^88^, ^89^ ^]^ Conversely, below the LCST, the polymer absorbs water and swells. This hydrogel‐based system holds promises for applications in soft actuators and drug delivery, where temperature‐triggered swelling or shrinking could modulate the release or capture of biomolecules. Shape memory polymers (SMPs) are another key class of thermo‐responsive materials, able to “remember” and return to a predetermined shape after deformation (Figure 3b).^[^ ^90^, ^91^ ^]^ SMPs maintain their permanent shape through chemical or physical crosslinking. When heated above their thermal phase transition temperature, these polymers significantly soften—experiencing a two‐to‐three order of magnitude decrease in elastic modulus—which allows them to be deformed into a temporary shape that can be fixed upon cooling. SMPs can be engineered from thermoplastic polymers like lactides, glycolides, and polyurethanes by adjusting molecular weight or incorporating block copolymers.^[^ ^78^, ^92^ ^]^ They have been applied in stents,^[^ ^93^ ^]^ drug delivery systems,^[^ ^94^ ^]^ tissue scaffolds,^[^ ^79^ ^]^ and orthopedic devices.^[^ ^95^ ^]^ In minimally invasive procedures, SMPs are often deformed into a compact form to reduce devices’ profile and enable implantation through small incisions.^[^ ^96^ ^]^ After placement, a specific stimulus triggers the polymer to revert to its original shape. Most biomedical SMPs are temperature‐responsive, with desired transition temperatures programmed at or below body temperature (37 °C). Polymers with higher transition temperatures would require external heating, which could pose safety risks as regulating temperature in devices without affecting adjacent tissues is difficult. Some SMPs, such as aliphatic polyesters, are designed to degrade into nontoxic by‐products through chemical or enzymatic hydrolysis in vivo after their functional period, making them suitable for resorbable implants and biodegradable sutures.^[^ ^80^, ^81^, ^97^ ^]^ Liquid crystal elastomers (LCEs) are polymer networks that combine the self‐assembly properties of liquid crystals with the elasticity of elastomers.^[^ ^98^, ^99^ ^]^ These materials contain rod‐like segments, called mesogens, embedded in the polymer chains. LCEs undergo large shape changes in response to stimuli like temperature and light due to the realignment of mesogens between ordered (nematic or smectic) and disordered (isotropic) phases (Figure 3b).^[^ ^100^, ^101^ ^]^ To control the magnitude and direction of strain, it is essential to align mesogens within the network film. When the alignment is uniform throughout the film, deformation occurs along the alignment direction of the mesogens. Such alignment can be achieved through techniques such as stretching, shearing, and the application of magnetic fields.^[^ ^102^, ^103^, ^104^ ^]^ Like SMPs, it is critical to engineer the trigger temperature of LCEs to be close to 37 °C for implant use.^[^ ^105^, ^106^, ^107^ ^]^

#### 2.1.3 pH‐Responsive Materials

pH‐responsive materials contain ionizable groups that accept or donate protons depending on the surrounding pH, causing changes in solubility, swelling, or conformation (Figure 3c).^[^ ^108^, ^109^ ^]^ These changes are typically reversible, allowing the material to revert to its original state when the pH shifts back to baseline.^[^ ^110^, ^111^, ^112^, ^113^, ^114^ ^]^ Since different body regions have distinct pH levels (e.g., the highly acidic stomach and the neutral bloodstream), these materials can be tailored for site‐specific applications. For instance, poly(acrylic acid) swells in basic environments, making it ideal for drug release in the intestines,^[^ ^115^, ^116^ ^]^ while chitosan is soluble in acidic conditions and gels in neutral to basic environments, which is useful for wound dressings, drug delivery, and tissue scaffolds.^[^ ^117^, ^118^, ^119^, ^120^, ^121^ ^]^ However, designing materials that respond precisely to small pH variations remains challenging, especially in applications requiring sensitivity to monitor pH changes without affecting healthy tissues.

### 2.2 On‐Demand Reversible Soft Actuation

The abovementioned functional materials can respond to biological conditions, which show promise in getting soft and conforming to the complex shapes of body tissues or transforming into a certain shape after implantation. While thermo‐responsive materials such as LCEs can be reversibly actuated, maintaining this reversibility in physiological environments is challenging, as the material tends to retain its shape after activation, and altering it without risking harm to surrounding tissues is difficult. In practice, this means that many “reversible” mechanisms described in Section 2.1 end up functioning more like single‐use or single‐transition actuators when implanted. However, in some cases, reprogrammable devices that show reversibility and repeatability in vivo are desired to perform more complex and dynamic tasks. Section 2.2 will therefore focus on on‐demand reversible actuation, leveraging techniques from cutting‐edge soft robotics to achieve truly repeatable shape changes in physiological conditions. Soft robotics, as one of the state‐of‐the‐art technologies, focuses on developing robots made from deformable materials.^[^ ^32^ ^]^ These robots, inspired by biological structures, can adapt to, and safely interact with dynamic environments, handle delicate objects, and navigate in confined spaces.^[^ ^60^ ^]^ In these systems, soft actuators—acting as “muscles”—generate the mechanical force needed for the functional movements of soft robots. Unlike traditional rigid actuators, soft actuators provide flexibility, biocompatibility, and adaptability to the body's natural movements, making them ideal for medical applications where interaction with soft tissues and organs is essential.

#### 2.2.1 Pneumatic Actuation

Pneumatic actuators, widely used in soft robotics, operate by pressuring air within chambers made from materials like silicone elastomers to drive movement (Figure 3d).^[^ ^32^, ^61^, ^122^, ^123^ ^]^ A similar mechanism is hydraulic actuation, where movement is achieved by controlling fluid pressure in soft chambers, offering higher force output due to the incompressibility of liquids.^[^ ^124^, ^125^ ^]^ Multi‐directional bending deformations can be achieved by selectively pressurizing parallel pneumatic chambers.^[^ ^126^ ^]^ Their simple design, high force output and the inherent softness of the materials make pneumatic actuators well‐suited for medical devices requiring precise and gentle interactions with tissues.^[^ ^127^ ^]^ For instance, integrating pneumatic actuators into catheters and endoscopes allows the devices to bend and navigate through narrow, tortuous pathways within the body like blood vessels or the gastrointestinal tract, which can minimize the risk of tissue damage that may occur with manual insertion.^[^ ^128^, ^129^, ^130^ ^]^ Additionally, they can be used in soft robotic surgical grippers to gently grasp or manipulate delicate tissues, where traditional rigid instruments might cause unintended injury.^[^ ^131^, ^132^ ^]^

#### 2.2.2 Tendon‐Driven Actuation

Inspired by biomechanics, where tendons transfer forces from muscles to bones to enable movement, tendon‐driven soft actuators are constructed by embedding cables—as tendons—within soft materials such as elastomers.^[^ ^133^ ^]^ Pulling or releasing the cables with motors enables the structure to bend or twist with precision and rapid response (Figure 3e).^[^ ^64^, ^65^ ^]^ To generate large torques, longer lever arms can be employed to amplify the moment for the same applied force.^[^ ^63^ ^]^ Reducing friction between the tendons and the structure materials can enhance output force by using low‐friction coatings like Teflon or applying biocompatible lubricants, such as silicone oil, within the channels. Additionally, both the tendons and the support structure must be sufficiently strong to withstand large forces. Similar to pneumatic actuators, tendon‐driven systems can be used in steerable catheters to access hard‐to‐reach areas during minimally invasive cardiovascular, urological, or neurological procedures, as well as in robotic‐assisted drug delivery systems for cancer treatment or neurological conditions.^[^ ^134^ ^]^ The external motor remains the primary obstacle to miniaturization, requiring a balance between output force and the size of auxiliary equipment.^[^ ^135^ ^]^

#### 2.2.3 Electrical Actuation

Electrical actuation is widely used for soft robotics. Its reliance on voltage input enables straightforward integration with bioelectronic implants without the need for auxiliary equipment. Here, we focus on two main types: dielectric elastomer actuators (DEAs) and ionic electroactive polymer actuators, which include ionic polymer metal composites (IPMCs) and conducting polymers (Figure 3f). These systems’ actuation states and amplitudes are controlled by modulating input power signal such as amplitude, frequency, and phase. DEAs consist of a dielectric elastomer sandwiched between two flexible electrodes, similar to the structure of an electric capacitor.^[^ ^66^, ^67^ ^]^ When a high potential is applied across the electrodes, electrostatic forces compress the elastomer in thickness and cause lateral expansion.^[^ ^68^ ^]^ Their muscle‐like actuation offers fast operation, high power density, mechanical compliance, and large strain capabilities.^[^ ^136^ ^]^ Recent progress has demonstrated their potential applications in cardiac assist devices to aid heart function or pump fluids within the body.^[^ ^137^ ^]^ However, DEAs typically require high activation voltages (in the kV range), which poses a challenge for implantable systems where power supply limitations and safety are concerns. Research is ongoing to reduce voltage requirements or develop materials with lower activation energy needs.^[^ ^138^, ^139^ ^]^

IPMCs consist of a polyelectrolyte membrane (a polymer that conducts ions, such as Nafion) sandwiched between two metal electrodes.^[^ ^70^ ^]^ When voltage is applied, hydrated cations in the membrane migrate, creating an asymmetric distribution of swelling that bends the actuator.^[^ ^140^, ^141^ ^]^ IPMCs operate at low voltages (typically under 10 V) and are highly flexible, making them a safer and more biocompatible choice for soft tissue interactions.^[^ ^142^ ^]^ Although IPMCs can achieve large deformations, they typically produce much lower actuation forces compared to DEAs or pneumatic actuators. Additionally, changes in hydration levels within the polymer over time may affect performance consistency, affecting their use in complex, dynamic environments. Conducting polymer actuators function through the reversible oxidation and reduction of conducting polymers like polypyrrole (PPy), polyaniline (PANI), and poly(3,4‐ethylenedioxythiophene) (PEDOT).^[^ ^143^ ^]^ These polymers undergo volume changes as ions and water molecules are inserted or expelled during the electrochemical process, resulting in mechanical movement.^[^ ^144^ ^]^ Free‐standing conducting polymer films exhibit linear deformation (either contraction or expansion) when electrically stimulated in a liquid electrolyte. When laminated with a passive layer, they can form bilayer actuators capable of bending in electrolytes, demonstrated in applications like active hinges, valves, and soft robotic grippers.^[^ ^69^, ^145^, ^146^ ^]^ Symmetric trilayer actuators, which consist of an ionically conductive membrane sandwiched between two conducting polymer layers (forming supercapacitor‐like electrochemical cells), allow actuation in air, which eliminate the need for liquid electrolytes and expand their range of applications.^[^ ^147^ ^]^ In general, ionic electroactive polymer actuators produce relatively low actuation forces and their speed are often limited by the ion diffusion process.

#### 2.2.4 Light‐Driven Actuation

Light‐responsive actuation employs smart materials responsive to light, such as hydrogels^[^ ^148^ ^]^ and liquid crystal polymers.^[^ ^149^ ^]^ These actuators are often composed of photoactive agents that are embedded in a soft matrix and can absorb photons and initiate mechanical actuation via photothermal or photochemical effects (Figure 3g).^[^ ^150^, ^151^, ^152^ ^]^ Carbon‐based nanomaterials, like carbon nanotubes, excel in photothermal conversion,^[^ ^153^ ^]^ while azobenzene demonstrates reversible photo‐isomerization between trans and cis states under ultraviolet light.^[^ ^154^ ^]^ By adjusting the light‐responsive components, actuators can be tailored to respond either across a broad spectrum^[^ ^155^ ^]^ or to specific wavelengths,^[^ ^72^ ^]^ allowing for selective and sequential movements. These actuators can mimic biological muscle functions, enabling tasks such as grasping delicate objects,^[^ ^73^, ^156^ ^]^ swimming,^[^ ^72^ ^]^ and other forms of locomotion.^[^ ^74^, ^157^ ^]^ A significant challenge for light‐responsive actuators in implantable devices is light's limited penetration through biological tissues. Visible and ultraviolet light have shallow penetration depths, confining actuators to near‐surface applications. In contrast, near‐infrared (NIR) light can penetrate tissues up to 3 cm,^[^ ^158^ ^]^ enabling wireless actuation deeper within the body. In addition, light stimuli could be modulated with high temporal and spatial resolution, allowing precise, remote, and localized control. For instance, by focusing light on a specific region of the actuator, it is possible to induce targeted bending, curling, or expansion within implants, thus minimizing impact on surrounding tissues or structures.

#### 2.2.5 Magnetic Actuation

Magnetic soft actuators are constructed by embedding magnetic particles, such as iron oxide or neodymium–iron–boron (NdFeB), within an elastomeric matrix. When exposed to an external magnetic field, the embedded particles experience a torque proportional to their magnetization, causing the composite to bend, twist, stretch, or compress (Figure 3h).^[^ ^35^ ^]^ Although the particles themselves typically do not rearrange significantly within the matrix, their magnetization drives the bulk deformation. Precise, localized control can be achieved by designing the spatial distribution of magnetic composites or by generating nonuniform magnetic fields through adjustments to electromagnetic systems or the movement of permanent magnets.^[^ ^159^ ^]^ Magnetic actuation offers key benefits such as biocompatibility and untethered remote control within hard‐to‐reach or enclosed anatomical regions. Because magnetic fields can penetrate biological tissues, this approach is especially useful for navigating challenging areas such as narrow blood vessels or complex tissue structures.^[^ ^160^ ^]^ For example, ferromagnetic soft robots shaped like catheters can travel through intricate cerebrovascular pathways,^[^ ^36^ ^]^ and wireless magnetic millirobots can be guided through distal cortical arteries, which are often inaccessible by traditional catheters.^[^ ^85^ ^]^ This minimally invasive approach can reduce infection risks, minimize tissue damage, and speed up patient recovery. Magnetic resonance imaging (MRI) has been used to track and control magnetic actuators within the vascular system, and for procedures like needle biopsy and tissue ablation.^[^ ^86^ ^]^ Nevertheless, one drawback is that specialized electromagnetic coils or bulky setups are necessary for generating and controlling the magnetic fields. Simplifying these control systems, achieving sufficient force and torque, ensuring precise localization, and optimizing the operational workspace for medical devices remain major challenges.^[^ ^161^ ^]^

#### 2.2.6 Acoustic Actuation

Acoustic actuation leverages the energy carried by sound waves, especially ultrasound, to induce movement, vibration, or deformation in materials or devices. Acoustic waves exert pressure on particles, droplets, or objects, and drive movement in the direction of wave propagation or toward regions of lower pressure (Figure 3i). This method is commonly used for particle manipulation, cell sorting, drug delivery, and microfluidic systems.^[^ ^77^, ^162^ ^]^ High‐intensity ultrasound can generate microbubbles that oscillate within structures, and produce localized forces for selectively manipulating microswimmers or other particles in liquids.^[^ ^163^, ^164^ ^]^ Since acoustic waves can travel through tissues, they enable non‐contact control of microrobots within the body.^[^ ^165^ ^]^ Despite their promise, one of the key challenges for in vivo applications lies in ensuring selectivity to avoid off‐target effects, such as unintended tissue stimulation, hyperthermic injury, or mechanical disruption. Ongoing strategies to mitigate this risk include tailoring the acoustic impedance of microrobots to make them responsive to specific frequencies,^[^ ^165^ ^]^ applying focused ultrasound to deliver energy precisely to selected regions,^[^ ^76^ ^]^ and using contrast agents or structural design to enhance their acoustic contrast with surrounding tissues.^[^ ^166^ ^]^ In addition, advanced imaging modalities can be used to visualize and monitor the actuation process in real time, enabling prompt detection and correction of any off‐target effects.^[^ ^75^ ^]^

## 3 Recent Advances in Shape‐Morphing Implants for Neural Interfaces

Despite progress in smart materials and soft robotics, their combination with microelectronics and in vivo actuation remains challenging. This difficulty mainly arises from the integration of stimuli‐responsive materials or components with existing electronics, where factors such as compatible manufacturing, strong mechanical adhesion, and the need for safe and precise actuation control within complex physiological environments must be carefully addressed. Actuators must generate sufficient force to overcome friction, capillary forces, and sometimes viscous resistance, all while maintaining structural integrity, and ensuring safety and effectiveness. The force output varies based on design, material properties, and the specific application. Therefore, a thorough understanding of each approach's strengths, constraints, and the specific requirements of the target in vivo site is crucial for optimizing soft actuators and robotic systems for practical biomedical use. In the following, we highlight recent breakthroughs in integrating smart materials and soft actuation with bioelectronics for minimally invasive neural implants and interfaces.

### 3.1 Softening Materials Enabling Accurate Positioning and Conformable Interfaces

**Figure 4:**
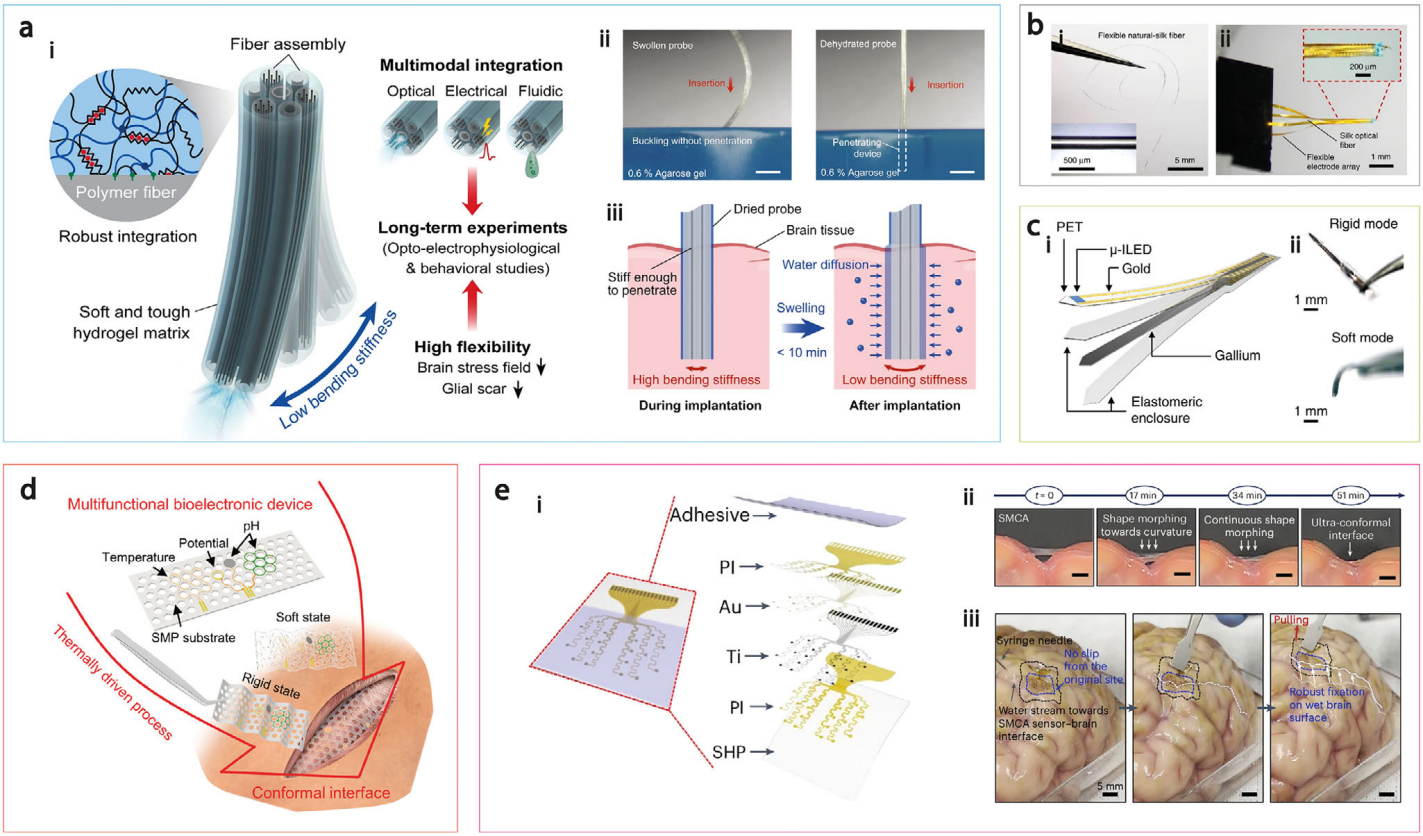
Softening implants. a) Multifunctional hydrogel hybrid probes. i) Conceptual illustration of the probe integrating optical, electrical, and fluidic functional fibers within a hydrogel matrix. ii) Insertion into a brain phantom: the swollen probe buckles, preventing penetration, while the dehydrated probe is stiff enough for easy insertion. iii) After implantation, the probe absorbs water from tissue, becoming compliant in its swollen state. b) Silk‐based self‐adaptive flexible neural probe. i) Natural silk optical fiber. ii) The probe combines an electrode array for electrophysiological recording and a silk optical fiber for intracranial light stimulation and easy insertion. c) Deep brain neural probe with tunable stiffness using gallium. i) Layout of the probe. ii) Probe in both rigid and soft modes. d) Multifunctional ECoG device with switchable rigidity and reconfigurable shapes. e) Stretchable cortex‐adhesive ECoG array. i) Layout of the device. ii) Shape morphing of the thin‐film device to conform to the wrinkled surface of the bovine cortex without external driving forces. iii) Demonstration of the adhesive interface's robustness to resist water flow and shear tension. Figure reproduced with permission from: (a), Reprod ref.,^[^ ^84^ ^]^ Copyright 2021, The Authors, Springer Nature; (b), ref.,^[^ ^83^ ^]^ Copyright 2022, The Authors, Springer Nature; (c), ref.,^[^ ^170^ ^]^ Copyright 2019, The Authors, some rights reserved; exclusive licensee American Association for the Advancement of Science; (d), ref.,^[^ ^171^ ^]^ Copyright 2023 The Authors, Advanced Electronic Materials published by Wiley‐VCH GmbH; (e), ref.,^[^ ^82^ ^]^ Copyright 2023, Springer Nature.

A promising alternative involves using stimuli‐responsive substrate materials with switchable stiffness, which are rigid enough to penetrate brain tissue and allow easy handling without additional rigid stiffeners, then softened once implanted to match the mechanical properties of brain tissue and adapt to the surrounding environment. To achieve these dynamic mechanical properties, implants must exhibit significant stiffness variation as they transition from external to internal conditions. As an example, compliant brain probes can be constructed by coating poly(acrylamide)‐alginate (PAAm‐Alg) hydrogels onto thermoplastic polymer‐based multifunctional fiber bundles (**Figure** **4**a).^[^ ^84^ ^]^ In their fully swollen state, these probes have low bending stiffness, which complicates direct insertion into deep brain sites. However, when dried, the probe's stiffness increases to 2.6 N m^−1^, allowing insertion without the need for guiding fixtures. After implantation, the hydrogel quickly absorbs water from surrounding tissues and the probe's stiffness is reduced to 0.42 N m^−1^. In addition to hydrogels, natural silk represents another excellent choice due to its exceptional mechanical strength and biocompatibility. Silk‐based devices (diameter 200–500 µm, length >200 mm) can actively transition from a hard, brittle state (elastic modulus 38.7 MPa, bending stiffness 3.05 × 10^−9^ N·m^2^) to a flexible one (elastic modulus 3.53 MPa, bending stiffness 2.77 × 10^−10^ N·m^2^) after hydration to minimize stress at the interface (Figure 4b).^[^ ^83^ ^]^ Notably, the softening of the gel is completed within 15 min of hydration, allowing a sufficient window for implantation while avoiding a prolonged rigid state. This adaptability can minimize tissue damage during implantation and improve long‐term biocompatibility, addressing issues like chronic foreign body responses and mechanical failure at the tissue/device interface. However, these materials also present certain challenges, particularly in ensuring predictable and stable swelling over time. The timing of the softening process must be carefully optimized, allowing sufficient time for successful implantation while avoiding a prolonged rigid state that could increase tissue damage. In addition, excessive swelling can lead to mechanical instability, while insufficient swelling may fail to achieve the necessary softening. The chemical composition and cross‐linking density of the polymer must be finely tuned to control the extent of swelling and softening. Additionally, the hydrogel coating volume must be carefully optimized—small enough to minimize footprint and tissue damage, yet large enough to modulate mechanical properties effectively. Achieving this balance requires careful material design for both surgical implantation and long‐term functionality post‐implantation.

Rigid implants are favored for their ease of handling during surgery, offering stability and precision in placement. In contrast, flexible microelectrodes provide superior conformability to irregular tissue surfaces, reducing mechanical mismatch and minimizing foreign body responses. However, accurately positioning flexible devices, such as intracortical and depth electrodes, is challenging due to their susceptibility to buckling instability under in‐plane compression. An ideal implant would combine the advantages of both—offering ease of surgical manipulation and precise positioning while ensuring long‐term integration with active tissues. Several strategies, including syringe injection,^[^ ^167^ ^]^ degradable reinforcing materials,^[^ ^168^ ^]^ and removable insertion shuttles,^[^ ^169^ ^]^ have been used to temporarily stiffen flexible devices for implantation.

Room temperature liquid metals, such as gallium (Ga) and its alloys, are another type of promising materials for softening implants due to their substantial change in rigidity. Gallium, with a melting point of 30 °C, can shift from an elastic modulus of 10 GPa in solid form to an effective stiffness in the tens of kilopascals—largely governed by oxide‐skin effects—once liquefied at body temperature. Neural probes incorporating Ga and polymeric substrates have shown reduced lesion size and decreased inflammatory glial responses compared to tungsten probes of similar dimensions (Figure 4c).^[^ ^170^, ^172^, ^173^ ^]^ Although some studies indicate that Ga‐based alloys exhibit relatively low toxicity compared to other metals,^[^ ^174^, ^175^ ^]^ comprehensive toxicological assessments remain in their early stages. Existing evidence suggests that factors such as the physical state, dosage, and surrounding environment all influence their toxicological behavior.^[^ ^176^ ^]^ Notably, the ionic radius of Ga closely matches that of iron (Fe), allowing it to interfere with Fe metabolism, affect the human immune system, and disrupt bacterial iron metabolism—an effect shown to have therapeutic benefits in mouse and human models of lung infection.^[^ ^177^, ^178^ ^]^ Therefore, the in vivo use of liquid metals requires rigorous caution, underscoring the necessity of hermetic encapsulation to ensure safety. Noncontact actuated implantation methods, such as magnetic^[^ ^179^ ^]^ and microfluidic actuation,^[^ ^180^ ^]^ have also shown potential for precise electrode positioning within the brain. Although these techniques may currently face limitations in actuation force and control systems, they hold significant potential and could become competitive with further optimization.

In addition to depth electrodes, the softening materials can be utilized in electrocorticogram (ECoG) devices. A multifunctional bioelectronic device with a shape memory polymer substrate has recently been demonstrated with switchable rigidity and reconfigurable shape.^[^ ^171^ ^]^ As the melting temperature of the shape memory polymer is close to body temperature, the device exhibited a dramatic reduction in modulus, from 100 MPa to 700 kPa upon implantation (Figure 4d). Animal studies confirmed its diagnostic potential, such as ECoG assisted by brain temperature monitoring and electrocardiograms aided by pericardial fluid pH measurement. Recently, Lee and colleagues reported a cortex‐adhesive sensor for closed‐loop transcranial ultrasound neurostimulation, where a viscoelastic hydrogel swells and fills in interfacial microvoid areas on the wrinkled cortex, thus forming a conformal and robust fixation (Figure 4e).^[^ ^82^ ^]^

### 3.2 Shape Morphing Peripheral Nerve Interfacing Implants

Due to limitations in fabrication techniques, bioelectronic devices are often produced in bulky, planar forms, making it difficult to form intimate interfaces with tissues or organs. While flexible, soft materials can provide some conformity through surgical interventions, achieving an ideal geometrical fit remains challenging for irregularly shaped or small tissues. Peripheral nerve stimulation, for example, holds significant clinical potential for treating conditions like chronic neuropathic pain, diabetes, and hypertension.^[^ ^181^, ^182^, ^183^ ^]^ However, current nerve cuffs are typically stiff, which complicates their application to the 3Dstructures of nerves, especially the small nerves of the autonomic nervous system. Soft peripheral nerve interfaces that use low‐modulus elastomers present one strategy for tackling these challenges.^[^ ^184^ ^]^ Recent work on “morphing electronics” provides a compelling approach to accommodate rapidly changing tissue geometries, as seen in pediatric and developmental contexts. The researchers demonstrated a multilayered design with viscoplastic electrodes and embedded strain sensors capable of reconfiguring and self‐healing during implantation, thereby reducing mechanical mismatch with nerve tissues that grow over time.^[^ ^185^ ^]^ Their system successfully maintained electrical stimulation and monitoring over a two‐month period in rat models, despite a 2.4‐fold increase in nerve diameter. Despite substantial academic progress, the emerging soft technologies remain at early technology readiness levels (TRLs) and must navigate complex regulatory approvals before achieving widespread clinical adoption.

**Figure 5:**
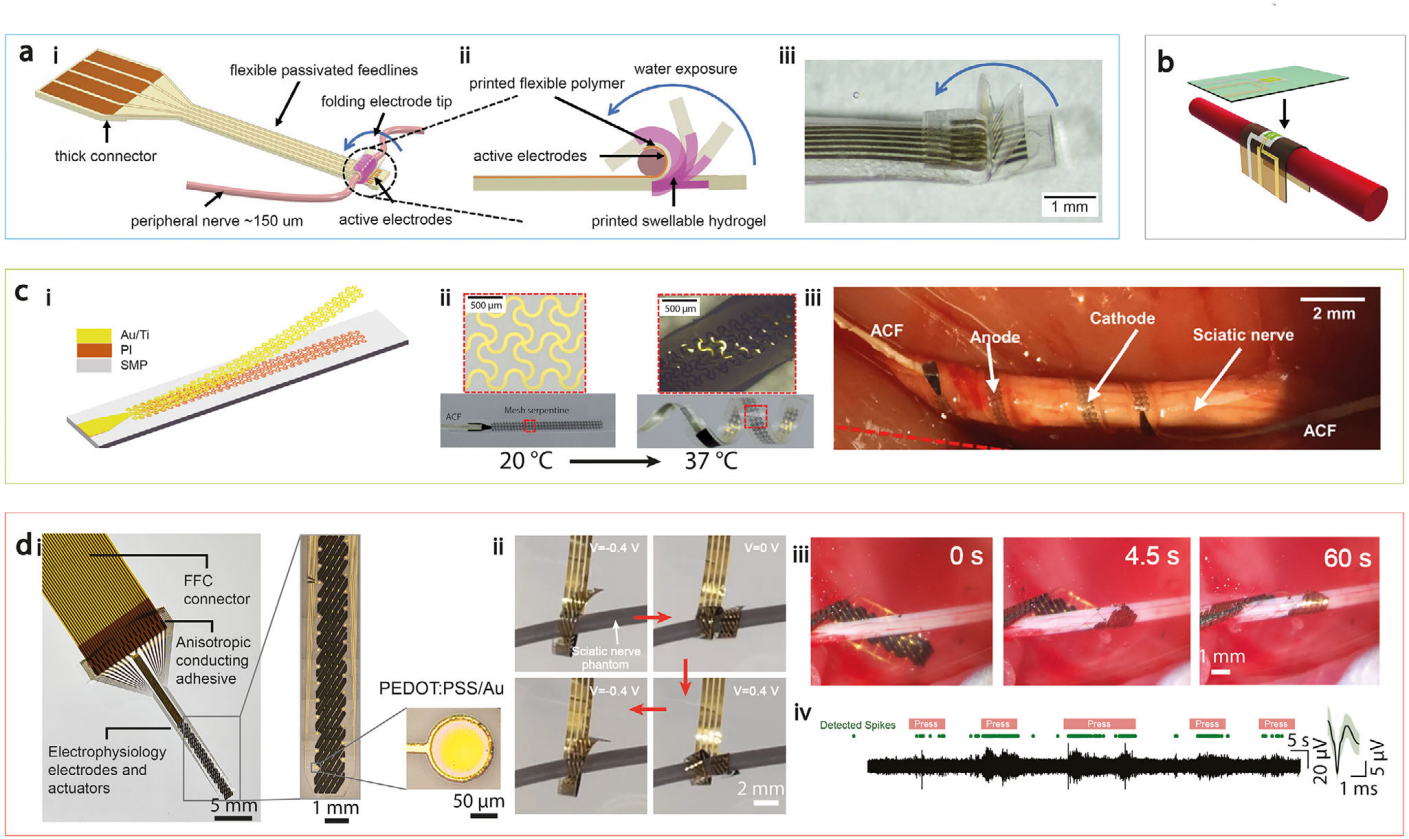
Shape morphing peripheral nerve cuffs. a) 3D printed, soft self‐folding electrode with a hydrogel‐based bilayer structure that swells to induce folding. i) Schematic of the whole cuff electrode. ii) Side‐view of the electrode tip at different stages during folding. iii) Perspective view of the folding process. b) Grasping cuff design utilizing a shape memory polymer substrate, integrated with an organic thin‐film transistor to detect small biological signals. c) Cuff electrode that wrap around nerves using shape memory effect triggered by body temperature. i) Layout of the device. ii) Temporarily flattened electrode transforms into a helical structure, through a shape memory effect triggered by body temperature. iii) Cuff electrode wrapping around a sciatic nerve for recording. d) Thin‐film‐based microelectrode arrays with reversible shape change for neural recording and removal. i) Structure of the thin‐film device, composed of conducting polymer‐based electrochemical actuators and electrophysiology electrodes. ii) Demonstration of the device wrapping around a 1.4 mm nerve phantom in PBS. iii) In vivo validation on a rat sciatic nerve, showing the cuff electrode forming a helical wrap by switching the voltage from −0.5 to 0 V. iv) Recorded signal from the sciatic nerve during hindpaw pressing. Figure reproduced with permission from: (a), ref.,^[^ ^186^ ^]^ Copyright 2023 The Authors. Advanced Materials published by Wiley‐VCH GmbH; (b), ref.,^[^ ^189^ ^]^ Copyright 2014 WILEY‐VCH Verlag GmbH & Co. KGaA, Weinheim; (c), ref.,^[^ ^190^ ^]^ Copyright 2019 The Authors, some rights reserved; exclusive licensee American Association for the Advancement of Science; (d), ref.,^[^ ^71^ ^]^ Copyright 2024, The Authors, Springer Nature.

Another issue with traditional cuff electrodes lies in their manual placement around nerves using forceps, hooks, or sutures, which increases the risk of nerve compression, trauma, and other complications. To address this challenge, integrating stimuli‐responsive materials and soft actuators into abovementioned soft nerve cuffs offers a promising solution. These materials can adapt dynamically to the body in ways similar to how living organisms respond to their environment through growth or movement.^[^ ^52^ ^]^ Recently, Hiendlmeier et al. proposed a 3D‐printed bilayer‐structured nerve cuff made from non‐swelling flexible material (polyurethane) and a highly swelling hydrogel (sodium acrylate).^[^ ^186^ ^]^ Upon contact with body fluids, the sodium acrylate swells, which exerts a force that causes the cuff to bend and fold around small nerves (100—200 µm in diameter) (**Figure** **5**a). Other materials, such as silk, have also shown large swelling in biofluids, excellent shape adaptability to nerves, and chronic biocompatibility.^[^ ^187^, ^188^ ^]^ Reeder et al. fabricated organic thin‐film transistors on a shape memory polymer, which can be implanted through a small slit and deployed into a 3D structure to grasp a cylindrical object, such as a nerve, with a radius of 2.25 mm (Figure 5b).^[^ ^189^ ^]^ Notably, while the shape memory polymer has a high glass transition temperature (70 °C), small water uptake (3%) at physiological conditions facilitates the plasticization of the polymer network and reduces the glass transition temperature to near body temperature, which allows the material to soften and adapt post‐implantation.

One of the main limitations of current cuff electrodes is their reliance on a split‐cylinder type design with fixed diameters, which makes it difficult to accommodate nerves of varying sizes. Peripheral nerves can vary significantly in diameter, sometimes by hundreds of micrometers, therefore pre‐sized cuff electrodes often fail to provide an optimal fit. A cuff that is too small or tight can exert excessive pressure, potentially causing injury over time, while an oversized cuff may attach too loosely, resulting in inadequate contact between the electrodes and the target nerve.^[^ ^191^ ^]^ To address this, Zhang et al. proposed a SMP‐based cuff electrode capable of self‐climbing onto nerves and forming 3D conformal neural interfaces, driven by body temperature.^[^ ^190^ ^]^ By leveraging the significant shift in elastic modulus from approximately 100 MPa–300 kPa across the phase transition, surgical implantation in confined spaces is greatly facilitated. This controllability allows the twining electrodes to be manipulated in a relatively stiff state for precise handling, then soften once positioned around the nerves. Animal studies demonstrated its effectiveness in vagus nerve stimulation to reduce heart rate and sciatic nerve recording (Figure 5c). However, once positioned, these shape‐adaptive cuffs cannot further adjust, restricting their ability to refine contact areas, optimize interfacing sites, or address signal degradation during long‐term use.

To overcome these limitations, incorporating soft robotic actuators with reversible and repeatable actuation is essential. Recent advances have integrated soft electrochemical actuators with thin‐film bioelectronics to create low‐voltage, shape‐actuated nerve cuffs.^[^ ^71^ ^]^ These actuators feature a bilayer design, with conducting polymer polypyrrole bonded to a passive parylene substrate (Figure 5d). The conducting polymer undergoes a reversible electrochemical process in biofluid, incorporating or ejecting ions and solvents, which leads to volumetric expansion or contraction and leads to controllable bending of the bilayer structure under voltages less than 1 V. By strategically designing the distribution of actuating elements during microfabrication, various shape transformations can be realized, such as bending to grasp a nerve or forming a helical structure that coils around the nerve in multiple loops. Additionally, through precise engineering of material thickness and distribution, these devices can maintain a default curling state, allowing them to self‐wrap around nerves when no voltage is applied. After recording, applying a small negative voltage (e.g. −0.5 V) gently loosens the interface, enabling easy extraction of the device. These adaptive cuff electrodes hold great promise for minimally invasive intraoperative nerve monitoring.^[^ ^192^ ^]^ Notably, many of these active approaches are still in early‐stage proof‐of‐concept demonstrations (i.e., low TRL), and continued research on durability, potential toxicity, sterilization procedures, and the user‐friendliness of actuation controls is needed.

### 3.3 Deployable Electrodes for Interfacing with the Central Nervous System

**Figure 6:**
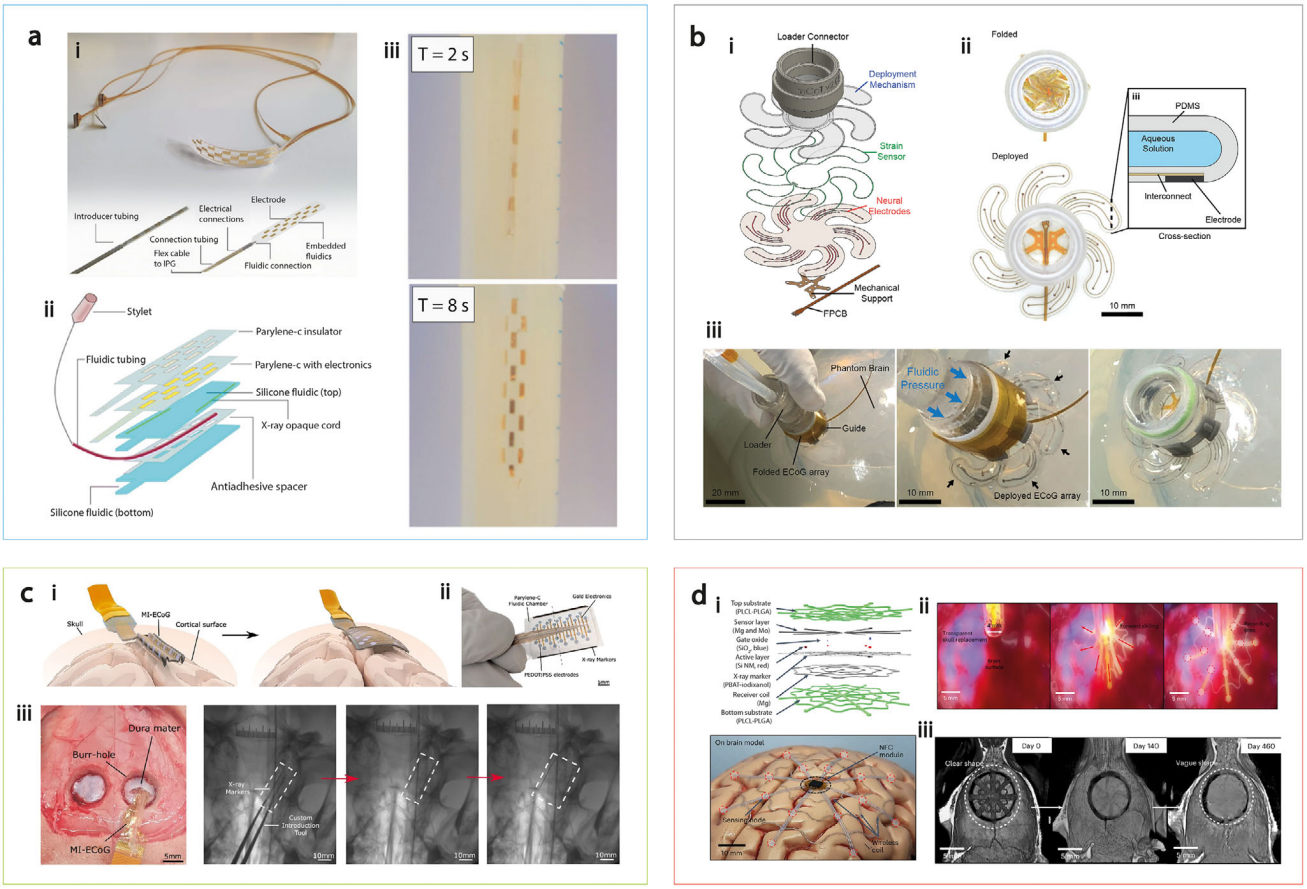
Deployable electrodes for minimally invasive interfaces spinal cord and brain interfaces. a) Spinal cord stimulating devices with shape actuation capabilities. i) Photograph of the device prior to packaging, showing thin film microelectronics and integrated fluidic channels and connections. ii) Exploded view illustrating details of the device architecture. iii) In vitro validation of shape actuation, where the device unrolls within seconds inside a water‐inflated latex balloon. b) Deployable multilegged soft ECoG array using pressure‐driven actuation. i) Image showing the device structure, consisting of the actuation component, strain sensor, and microelectrodes. ii) Top view of the ECoG array in its folded (top) and deployed (bottom) states, actuated by a positive fluidic pressure differential. iii) Deployment in a phantom brain model. The device is implanted through a 22 mm diameter burr hole, and then everted via fluidic pressure actuation. c) Minimally invasive, large‐area ECoG arrays enabled by soft fluidic actuation. i) Illustration of the device's working mechanism: folded for implantation through a burr‐hole craniotomy, followed by in situ expansion for large‐area coverage. ii) Photograph of the fabricated thin‐film device before folding. iii) In vivo validation on a porcine cortex, with craniotomy for implantation and X‐ray images showing device deployment (outlined in white boxes). d) Biodegradable, self‐deployable ECoG electrode. i) Exploded view of the device, featuring biodegradable shape memory polymers for shape recovery and encapsulation, an X‐ray marker for imaging, and biodegradable electrodes and sensors. ii) In vivo validation of the deployment process in a canine head, using a transparent skull replica with fluorescence imaging. iii) Micro‐CT images of a rat's head over 460 days post‐implantation, showing gradual degradation of the device, with near‐complete disappearance by day 460. Figure reproduced with permission from: (a), ref.,^[^ ^62^ ^]^ Copyright 2021 The Authors, some rights reserved; exclusive licensee American Association for the Advancement of Science; (b) ref.,^[^ ^37^ ^]^ Copyright 2023 The Authors, some rights reserved; exclusive licensee American Association for the Advancement of Science; (c) ref.,^[^ ^31^ ^]^ Copyright 2024, The Authors, Springer Nature; (d) ref.,^[^ ^193^ ^]^ Copyright 2024, Springer Nature.

There is a significant trade‐off between invasiveness and functionality in neural interfacing devices that requires a delicate balance. For example, electroencephalogram (EEG) devices can be attached to the scalp noninvasively, but they only capture general brain activities from broad regions and could miss fine and critical neural details. In contrast, implanted devices like intracortical electrodes can detect and stimulate individual neurons, enabling precise control in applications like prosthetics and brain‐machine interfaces. Similarly, due to the limitations of current spinal cord stimulation (SCS) technologies, clinicians and patients must choose between bulky paddle‐type electrodes, which offer better spatial coverage but require invasive surgery, and percutaneous lead‐type electrodes, which are easier to implant but suffer from limited coverage and electrode migration. To address this issue, Woodington et al. introduced a minimally invasive, shape‐actuated SCS device using soft actuators.^[^ ^62^ ^]^ Fabricated via photo‐ and soft lithography techniques, the device incorporates thin, flexible electronics and fluidic channels for actuation (**Figure** **6**a). Designed to fit inside a 14‐gauge Tuohy needle, it remains under 2 mm in diameter, including all packaging, and is stabilized during insertion by a 400‐µm stylet to prevent kinking. Finite element modeling suggests that a pneumatic pressure of 2kPa is sufficient to deploy the devices, while the failure threshold remains significantly higher, ≈8–12 kPa. Electrical connections are made via a custom flexible cable bonded with anisotropic conductive film. Once implanted, the device expands to form paddle‐type interfaces, offering a large, conformal footprint while minimizing surgical risk.

In brain interfaces, electrocorticography (ECoG) is widely used for monitoring brain activity, particularly in the localization of epileptogenic zones, functional brain mapping, and during brain tumor surgery. Traditional ECoG grids are large and require extensive craniotomies to expose the cortex over areas at least as large as the size of the devices, which carry risks like infection and brain damage. While smaller implants are available, they offer limited cortical coverage, creating the need for devices that balance minimal invasiveness with large‐area coverage. Recently, large‐area soft robotic electrode arrays deployable through small burr holes have been developed. These systems use a mechanism called eversion, where initially folded soft legs extend under fluid pressure to unfold and cover the brain's surface (Figure 6b).^[^ ^37^ ^]^ During deployment, each leg inflates over approximately 30–40 s, requiring pressures on the order of several kilopascals. This deployment pressure is highly dependent on design parameters, including leg width, taper angle, and radius of curvature. By reducing the thickness of the soft substrate, the required deployment pressure can be further decreased, thereby minimizing brain indentation during implantation. In addition to the eversion module, the devices integrate microelectrodes and strain sensors to enable real‐time monitoring during deployment. In a proof‐of‐concept study with a minipig model, these arrays successfully recorded sensory cortical activity, and could be easily pulled out from the brain after use.

Coles et al. drew inspiration from origami and designed large‐area ECoG implants that can change shape via fluidic actuation.^[^ ^31^ ^]^ The device is folded in a concertina pattern, allowing it to be compactly packaged. Upon deployment, fluidic actuation overcomes friction between the device and the arachnoid membrane, applying pressure to slightly depress the brain while expanding within the virtual subdural space to achieve lateral coverage (Figure 6c). A constant air pressure of 16–17 kPa was manually applied with a syringe to achieve full in vitro expansion, representing less than half the maximum pressure these devices can withstand (34–37 kPa for air, and 77–89 kPa for water). Bae et al. introduced a biodegradable, self‐deployable electronic tent electrode for cortical interfacing.^[^ ^193^ ^]^ This system can be packaged in a syringe to deliver through a small hole, and then self‐deploy by leveraging the shape‐memory characteristics of a poly(lactide‐co‐ε‐caprolactone)‐poly(lactic‐co‐glycolic acid) (PLCL‐PLGA) substrate (Figure 6d). The device can expand approximately 200 times its initial size to cover a large interfacing area. Additionally, it incorporates multiplexing arrays and a wireless module for near‐field communication and data transfer. The electrode naturally decomposes within the body after use, reducing complications related to tethering and eliminating the need for surgical removal. Shape memory alloys, such as nitinol with a phase transition temperature adjusted to body temperature, could also achieve similar actuation during surgery.^[^ ^194^ ^]^

### 3.4 3D Microelectrode Arrays (MEAs) for Organoids and Spheroids

The demand for 3D and shape‐adaptable designs extends beyond implantable neural interfaces to the precise and comprehensive monitoring and modulation of 3D in vitro biosystems. Among these systems, organoids and spheroids stand out as pioneering 3D cell culture models that offer versatile platforms for exploring human biology, enabling deeper insights into cellular behavior, tissue development, and disease mechanisms. Organoids are lab‐grown, miniature models derived from stem cells that self‐organize into 3D structures, which closely resemble, at least in part, the architecture and function of human organs such as the brain, liver, and lungs.^[^ ^195^, ^196^, ^197^ ^]^ Spheroids, while typically simpler and often lacking the full cellular complexity of organoids, remain valuable for studying fundamental cellular interactions and drug responses. In neuroscience and neuroengineering, brain organoids are especially invaluable for replicating critical features of the human brain, such as layered cortical structures and neural connectivity. In addition, these models can reproduce functional abnormalities, providing unique opportunities to investigate neurological conditions such as epilepsy and neurodegenerative diseases.

Electrophysiology is essential for studying brain organoids, as it provides direct access to electrical activity that dominates neural function. Due to their complex, neuron‐rich composition, brain organoids exhibit spontaneous electrical activity and network formation that mimics early brain development. MEA technology allows us to capture this activity in real time, revealing how neurons communicate and organize into functional circuits.^[^ ^198^ ^]^ However, conventional planar MEA platforms have limited interface areas, typically confined to the bottom of the 3D tissue structures, restricting the capture of comprehensive neural signals across the entire structure. Shape‐morphing bioelectronics, by contrast, conform to the surface of 3D assemblies, providing a larger interfacing area and enabling full mapping of electrical signals and their propagation across these biosystems. These bioelectronics can adapt to natural growth or embed partially within organoids and spheroids to provide stable, high‐resolution data over extended periods. This adaptive interfacing opens new possibilities in neuroscience, allowing for detailed studies of cellular dynamics, network connectivity, and treatment responses in organoids and spheroids. Such advances are crucial for modeling neurological diseases, testing drugs, and advancing personalized neurotherapeutic approaches.

**Figure 7:**
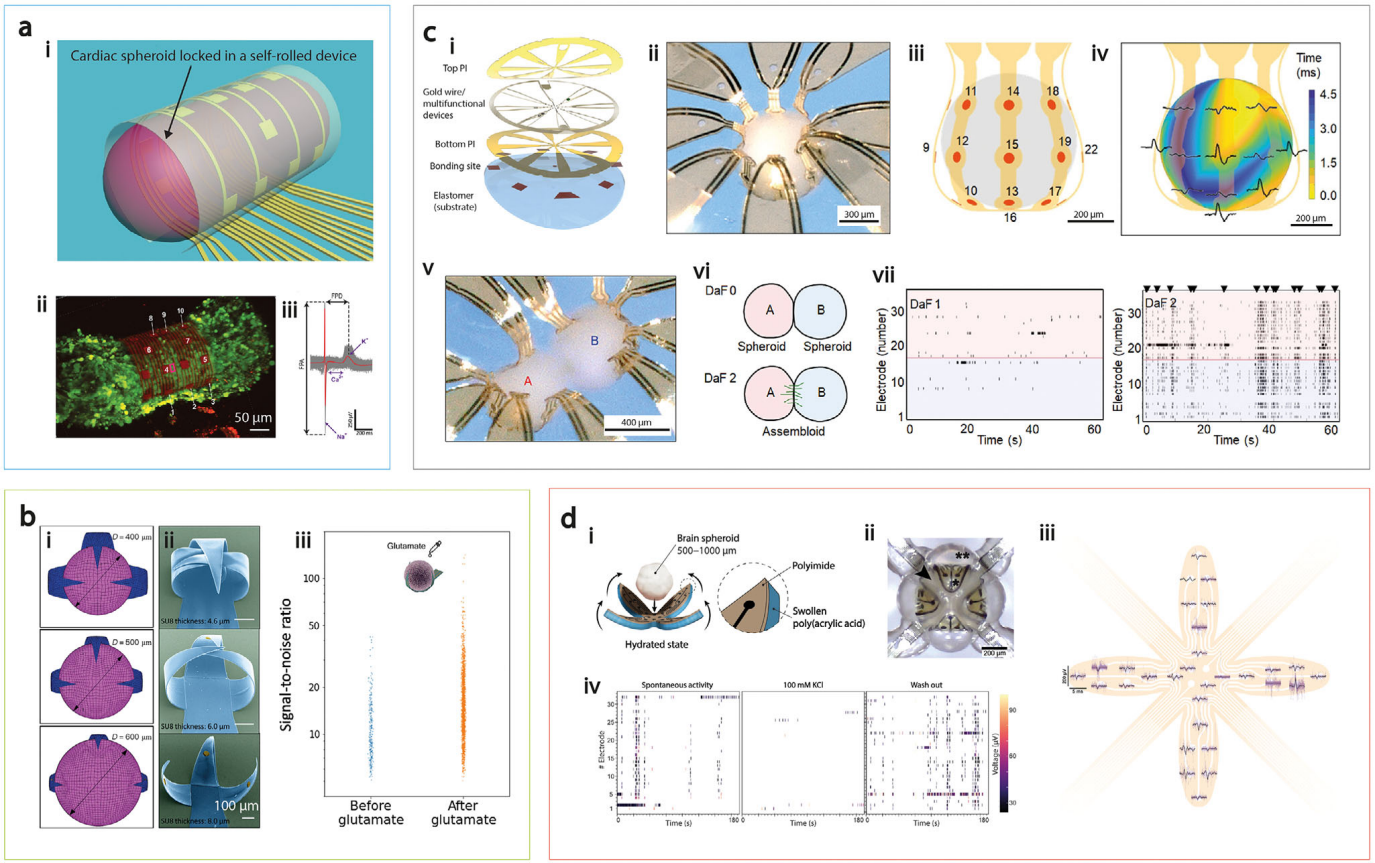
Advanced 3D MEAs for interfaces with organoids and spheroids. a) Self‐rolled biosensor array for electrical measurements of human electrogenic spheroids. i) Schematic of a cardiac spheroid encapsulated in a self‐rolled device for 3D electrical measurements. ii) 3D confocal microscopy image of a cardiac spheroid labeled with Ca^2+^ indicator dye. iii) Averaged field potential peak (red trace) alongside raw data (gray traces). b) 3D shell MEAs for interfacing with brain organoids. i) Finite element analysis showing the device's adaptation to organoids of various sizes. ii) Scanning electron microscopy images illustrating shell electrodes with different folding curvatures. iii) Spike distribution from a brain organoid recorded before and after glutamate treatment. c) 3D multifunctional neural interfaces for cortical spheroids and assembloids. i) Exploded view of the device's layered structure. ii) Optical image of a cortical spheroid enclosed in a 3D device. iii) Illustration showing the distribution of microelectrodes across the spheroid surface. iv) Representative recordings of the 3D spatial propagation of wave spreading, firing, and bursting events across the spheroid. v) Optical image of a 3D device connecting two neural spheroids. vi) Illustration showing the functional assembly of a pair of spheroids. vii) Raster plots from Day 1 (DaF 1) and Day 2 (DaF 2) after infusion, showing asynchronous firing on Day 1 and synchronized bursting and enhanced activities on Day 2. d) Hydrogel‐actuated 3D MEA for brain spheroid electrophysiology. i) Schematic showing the device's transformation from 2D to 3D, driven by the swelling of a poly(acrylic acid) hydrogel. ii) Microscopy image of the e‐Flower device enveloping a spheroid. iii) Overlaid field potential waveforms detected by the electrodes in a 5‐min recording. iv) Raster plots depicting neural events detected by the 3D MEAs before, during, and after exposure to 100 mM KCl. Figure reproduced with permission from: (a), ref.,^[^ ^199^ ^]^ Copyright 2019 The Authors, some rights reserved; exclusive licensee American Association for the Advancement of Science; (b) ref.,^[^ ^200^ ^]^ Copyright 2022 The Authors, some rights reserved; exclusive licensee American Association for the Advancement of Science. (c), ref.,^[^ ^201^ ^]^ Copyright 2022 The Authors, some rights reserved; exclusive licensee American Association for the Advancement of Science; (d), ref.,^[^ ^202^ ^]^ Copyright 2024 The Authors, some rights reserved; exclusive licensee American Association for the Advancement of Science.

For instance, Kalmykov et al. developed a self‐rolled sensor array for electrophysiological measurements of spheroids to investigate cell‐cell communication within complex cellular assemblies.^[^ ^199^ ^]^ In this device, residual stress between metal films, induced during fabrication, causes the array to roll into a tubular structure that envelops the spheroid (**Figure** **7**a). Controllable rolling can be achieved by adjusting metal deposition parameters and film thickness. The device's high spatial resolution enabled recording from individual cells within the 3D spheroid, while its temporal precision captured ionic currents (Na^+^, K^+^, Ca^2+^) with a high signal‐to‐noise ratio. This spatiotemporal resolution allows for 3D signal propagation mapping, a feat that surpasses the limitations of Ca^2+^ transient imaging, which is restricted to individual planes. Similarly, Huang et al. have developed miniaturized MEA shells with self‐folding polymer leaflets for recording and stimulating brain organoid activity.^[^ ^200^ ^]^ This design employs a self‐folding negative photoresist polymer bilayer, with folding tunability based on thickness and exposure energy, guided by finite element modeling (Figure 7b). These customizable MEA shells can be adapted to organoids of varied sizes. The researchers compared the cumulative firing rates and electrical responses of brain organoids in 2D and 3D recordings when exposed to glutamate, observing that 3D MEAs detected both an increase in firing rate and a more pronounced glutamate‐mediated response.

In another work, a 3D multifunctional spheroid interface was developed using prestress from pre‐strained elastic substrates.^[^ ^201^ ^]^ This platform enabled the detection of the spreading of coordinated bursting events across cortical spheroid surfaces and the measurement of processes associated with formation and regrowth of bridging tissues across a pair of such spheroids (Figure 7c). Most recently, a flower‐shaped MEA (“e‐Flower”) was developed, capable of enveloping brain spheroids through shape‐morphing triggered by the addition of cell culture medium (Figure 7d).^[^ ^202^ ^]^ The swelling properties of a polyacrylic acid hydrogel grafted to a polyimide substrate induce the bending, and the bending curvature can be tuned by adjusting the reswelling medium and hydrogel cross‐linker concentration, achieving a minimum curvature of 300 µm in minutes. This device captures spontaneous neural activity across the spheroid surface, offering the potential for comprehensive neural signal recording.

## 4 Current Challenges and Future Work

Although the convergence of intelligent systems and bioelectronics has been demonstrated, the direction is still in its early stages, with numerous challenges hindering large‐scale deployment and clinical acceptance. Accelerating progress requires close collaboration among materials scientists, roboticists, surgeons, biomedical scientists, and engineers to tackle practical issues and overcome these barriers.

### 4.1 Design Considerations for the Innovative Use of Soft Actuation in Bioelectronics

Integrating soft actuation technologies into bioelectronic implants can significantly improve device placement and promote adaptive, conformable, and deployable interfaces with tissue. These technologies offer mechanical flexibility and can navigate complex physiological environments. A common strategy to explore and enhance these possibilities involves adapting existing actuation and robotic methods. However, this path is fraught with challenges, such as actuation limitations and safety issues, that must be carefully addressed during design and development.

#### 4.1.1 Actuation Mechanism Selection

Many actuation methods face significant challenges in vivo. Smart materials responsive to physiological conditions lose actuation capability after a single use, posing problems for devices requiring repetitive actuation to adjust their behavior over time. Externally controlled methods like electrical, magnetic, or acoustic actuation may be more reliable for repetitive use. Pneumatic actuators are highly compliant, easy to fabricate, and provide large deformations, but precise control is challenging due to the nonlinear and viscoelastic nature of elastomers.^[^ ^203^ ^]^ Their dependence on external pumps poses a challenge for miniaturization. When using dielectric elastomer actuators, issues like dielectric breakdown and encapsulation defects must be addressed, and operating voltage reduced for safety. Electrothermal actuators suffer from low efficiency and frequency due to slow heating and cooling cycles. Sustained or repetitive thermal stimulation cycles in the body can pose a great risk of tissue damage, while light‐triggered actuation is limited by light's shallow tissue penetration. A thorough understanding of the advantages and limitations of available actuation strategies, along with the specific requirements of each application, is essential for optimizing design and functionality. This is especially important in implantable technologies, where output performance, biocompatibility, and safety are paramount (Table 1). Hybrid actuators, combining two or more of these mechanisms, may offer unique actuation capabilities beyond the simple sum of individual techniques. For example, a magnetically navigable catheter that moves through tortuous vascular pathways could integrate pneumatic or electrical actuation for localized tissue manipulation, drug delivery, or electrode deployment. Although this approach may require two control systems, which is a significant limitation, the potential for precise, minimally invasive guidance coupled with on‐demand shape changes can be advantageous in specialized medical scenarios, such as endovascular^[^ ^204^ ^]^ and endocisternal^[^ ^205^ ^]^ neural interfaces or targeted drug delivery in inaccessible brain regions.^[^ ^206^ ^]^ Nonetheless, the complexity introduced by hybrid actuation must be carefully justified against the demands of each application, balancing the need for advanced functionality with the practicalities of device fabrication, control, and clinical safety.

#### 4.1.2 Precise Actuation

Precise actuation is crucial to navigate in complex biological environments, and avoid damaging healthy tissue, especially when interfacing with delicate structures. This can be realized by optimizing device design and control systems. For example, to overcome the reliance of soft pneumatic actuators on hard valves for airflow controlling, a bistable membrane can be exploited to create entirely soft valves, which would allow autonomous feedback and feedforward control.^[^ ^203^ ^]^ The incorporation of artificial intelligence (AI) could play a significant role in improving actuation control and adaptability. AI‐assisted systems could enhance actuation control and adaptability, enabling fine‐tuning in response to dynamic physiological conditions and improving the safety and effectiveness of bioelectronic devices. Imaging compatibility is necessary for responsive bioelectronic devices to ensure visibility and compatibility with surgical imaging and guidance techniques like magnetic resonance imaging (MRI), computed tomography (CT) scanning, and fluoroscopy. Integrating mechanically flexible X‐ray markers into conformable devices may be necessary, as thin‐film electrodes alone do not provide sufficient attenuation to be visible under fluoroscopy.^[^ ^207^ ^]^ Additionally, certain materials like metals or electronics are not compatible with MRI; the strong magnetic fields can interfere with or damage such objects, and these materials can cause artifacts in the images.

#### 4.1.3 Scaling Down, Fabrication and Robustness

Scaling down large soft actuators for biomedical applications is not a straightforward process. Traditional soft actuators are often fabricated using molding techniques, with critical components such as strong fibers or embedded control elements added manually. However, these fabrication methods are difficult to apply at sub‐centimeter or millimeter scales needed for implants. Rescaling requires careful consideration of multiple factors such as mechanical properties, weight‐bearing capacity, and integration of critical elements. As actuators become thinner, softer, and more fragile, they can bear only minimal additional weight. Creating untethered soft robotic devices further complicates matters because the low force and power density typical of soft actuators limits their ability to carry their own power supplies and control electronics. Nonetheless, notable progress have been achieved. For instance, Ji et al. have developed sub‐gram, autonomous fast soft robotic insects powered by DEAs operating below 500 V.^[^ ^208^ ^]^ Translating such breakthrough to implantable bioelectronic applications would be highly promising if safety issues could be solved. Meanwhile, advances in 3D printing,^[^ ^209^, ^210^ ^]^ laser micromachining,^[^ ^211^, ^212^, ^213^, ^214^ ^]^ and lithography^[^ ^215^ ^]^ have facilitated the integration of multiple materials with enhanced consistency, enabling the fabrication of sophisticated, miniaturized soft actuators. These technologies allow simultaneous manufacturing of both actuators and microelectronics with exceptional precision and reproducibility, ensuring compatibility between the two systems. The employed techniques must align with industrial‐scale manufacturing environments, meaning that the required tooling and equipment should be cost‐effective, reproducible, and amenable to strict quality control. However, achieving the throughput necessary for mass production—and at a realistic cost point—remains a challenge. Innovative and cost‐effective strategies that simplify the fabrication process without compromising precision or fidelity are still highly needed for large‐scale production. In addition, hermetic encapsulation technologies offering low permeability to biofluids and better chemical/physical compatibility with biological tissues are necessary to safeguard the device over extended periods.^[^ ^216^ ^]^ In a dynamic, wet physiological environment, the adhesion between different materials is critical for long‐term durability. Improving device robustness is equally important, since failure may occur under repeated manipulation during fabrication, packaging, and final use. Special attention is warranted at the connection points between soft devices and external rigid controllers to enhance durability. Actuation reliability and reproducibility are also essential. For instance, fluidic actuation devices may require relatively high pressure for in vivo manipulation, where even small pinholes or tears in the polymeric chamber could render the devices non‐functional. Optimizing the fabrication process can further increase device yield and consistency. Additionally, the added weight of bioelectronics can alter the device's mechanical properties and actuation modes, highlighting the need to consider these factors during the initial design phase for seamless system integration. Finally, embedded microelectrodes must remain functional under large, repetitive deformations associated with actuation to ensure consistent performance over time.

#### 4.1.4 Embedding Physical Intelligence

Embedding physical intelligence into materials and structures enables complex actions. The key condition for shape programming is introducing asymmetry or inhomogeneity into materials to define default deformation and control local actuation. This can be achieved through methods such as crosslinking gradients, patterning of stacked layers, introducing holes or cuts, or inducing pre‐strain. For example, inducing pre‐strain in pneumatic actuators would allow zero‐power holding and increase stored elastic strain energy, which enables their rapid and programmable actuation and recovery.^[^ ^217^ ^]^ Ultraviolet‐introduced crosslinking of selected regions of a gel leads to differences in local swelling, thus creating surfaces with constant Gaussian curvature (spherical caps, saddles, and cones), zero mean curvature (Enneper's surfaces), and more complex, nearly closed shapes.^[^ ^218^ ^]^ Origami and kirigami techniques can be incorporated with responsive materials or applied stimuli to achieve stiffness manipulation and motion programming of spatial structures within confined spaces. Applying kirigami principles to actuator surfaces can introduce directional friction effects.,^[^ ^219^ ^]^ and embedding strategically designed kirigami sheets into elastomeric membrane can transform inflatables into various complex shapes.^[^ ^220^ ^]^ Additionally, programming the sequence of deformation is essential for achieving complex and multiple shape configurations. For example, hinges responsive to external light of different wavelengths can be integrated to realize sequential self‐folding of 2D polymer sheets into 3Dobjects.^[^ ^221^ ^]^ The actuation rates of responsive materials can be optimized using strategies such as snap‐buckling instabilities, which provide significantly faster responses compared to the typically slower mechanisms like gel swelling.^[^ ^222^ ^]^ To design soft actuators capable of adapting to diverse tasks and environments, scientists and engineers are encouraged to draw inspiration from nature. For instance, soft devices may emulate plant growth, fish swimming, or worm‐like self‐propulsion. By mimicking these biological strategies, engineered actuators could achieve enhanced flexibility, adaptability, and functionality in harsh biological environment. In the applications of implantable electrodes, embedding physical intelligence into functional materials could allow the devices to sense and adapt to various physiological cues—such as local pressure changes, biochemical gradients (e.g., pH, oxygen levels, or biomarkers), or mechanical perturbations from tissue movements. By actively responding to these signals (e.g., by altering shape, stiffness, or contact force), the electrodes might optimize the interaction between devices and tissue, reduce mechanical stress on surrounding tissues, or stabilize electrode placement to maintain consistent stimulation or recording. In this way, complex actuation may support more targeted therapies, for example, dynamic gripping around a peripheral nerve only when needed, or controlled release of anti‐inflammatory agents in response to a localized rise in inflammatory markers. Such capabilities could help extend device lifetime, reduce complications, and improve clinical outcomes compared to static or manually adjusted implants.

#### 4.1.5 Limitations of Laboratory Demonstrations

Despite the surge in studies on responsive materials, actuators, and soft robotics, most demonstrations occur in controlled laboratory settings, such as dry, hard and static lab surfaces or Petri dishes, which are far different from the complex physiological environment. The human body presents wet, dynamic, soft, temperature‐controlled, and confined spaces with various resistive forces that challenge actuation. While current soft actuators can move easily in air or Newtonian liquids, they may struggle against in vivo resistive forces like friction, pulsatile biofluid flow, capillary forces, and tissue compression. For example, the non‐Newtonian properties and high‐speed pulsatile flow of blood can hinder miniaturized robots in the cardiovascular system. Moreover, most soft robots lack sufficient force output to operate effectively in delicate tissues like the brain. Researchers must therefore test their responsive materials and mechanisms in more physiologically relevant settings to validate their credibility.

### 4.2 Multi‐Functional Design

Electrodes for recording and stimulation of electrically active tissues are a critical feature for all implantable bioelectronic devices. Additional features—such as optoelectronic and microfluidic elements—can be integrated to enhance the versatility and therapeutic potential of these devices.^[^ ^223^, ^224^, ^225^ ^]^ Such multi‐functional design can improve performance, enabling more comprehensive monitoring, precise control, and targeted therapeutic intervention.^[^ ^226^ ^]^ However, achieving this integration requires sophisticated designs in materials, structures, and functions—not merely connecting components with wires or circuits—and often necessitates advanced manufacturing techniques to create seamless and biocompatible interfaces.^[^ ^227^, ^228^ ^]^

#### 4.2.1 Mechanical Feedback and Distributed Sensing

Mechanical sensing is a critical function that can be integrated into bioelectronic systems. Real‐time feedback on mechanical deformation ensures precise control of soft actuators, allowing the system to adjust actuation and prevent excessive force that could damage tissue. This feedback is especially important in applications requiring delicate interactions with soft tissue.^[^ ^229^, ^230^ ^]^ In addition to mechanical sensors, multimodal sensors—such as those for pH and temperature—can monitor physiological conditions at the implant site.^[^ ^231^, ^232^, ^233^ ^]^ Changes in local pH or temperature may indicate infection, inflammation, or other adverse responses, allowing the system to react appropriately by adjusting actuation parameters or initiating therapeutic interventions. Developing smart materials that perform multiple functions simultaneously is another opportunity. For example, shape‐memory polymers or hydrogels that change properties in response to environmental stimuli could provide both actuation and therapeutic release, enabling bioelectronic devices that autonomously adapt to physiological conditions. Bioelectronic devices often have varying physical properties (e.g., flexibility, stiffness, conductivity) across different regions due to their diverse materials and components. This heterogeneity makes it difficult to collect accurate feedback from a single sensor. Therefore, distributed sensors across the device are desirable. By collecting data from multiple locations, the system can generate a comprehensive map of mechanical and environmental conditions, providing a more accurate representation of device‐tissue interactions, reducing the risk of unintended tissue damage, and enhancing overall performance. However, significant challenges persist in developing combinations of active and passive materials that meet all the requirements for robustness, biocompatibility, fabrication, and function.

#### 4.2.2 Drug and Cell Delivery

Integrating drug delivery systems into shape‐adaptive bioelectronic devices is another promising avenue for multifunctional design. Combining actuation and sensing with controlled release of therapeutic agents creates a powerful platform for treatment and diagnostics. For example, an implanted device could monitor tissue conditions, detect inflammation, and automatically release anti‐inflammatory drugs to manage immune responses.^[^ ^234^ ^]^ Similarly, bioelectronic implants could deliver drugs that promote healing or prevent infection in the early postoperative period, addressing critical challenges in implantable devices.^[^ ^235^ ^]^ Recent advances in organic bioelectronics have also demonstrated the ability to selectively deliver neurotransmitters and other bioactive compounds through “iontronic” components both in vitro and in vivo.^[^ ^236^, ^237^ ^]^ Integrating cell delivery mechanisms into bioelectronic devices could enable regenerative medicine applications.^[^ ^238^, ^239^ ^]^ Soft actuators with embedded microfluidic channels could deliver stem cells or other therapeutic cells to damaged tissues, facilitating repair or regeneration.^[^ ^240^ ^]^ In neurological applications, bioelectronic devices could not only stimulate neurons but also deliver neural stem cells to promote recovery. Combining these therapeutic capabilities with real‐time monitoring could significantly improve patient outcomes by ensuring interventions are precisely timed and targeted. However, the inclusion of multiple functions—such as sensing, actuation, drug and cell delivery—places significant demands on the device's power supply and control systems.

### 4.3 Untethered Implantable Actuation Systems

Tethered actuation systems rely on physical connections—such as electrical wires or fluidic tubes—for power delivery, control signals, or fluids. While common in research and medical applications due to their simplicity, high force output, and agile deformation capabilities, they pose significant challenges when interfacing with the body, especially in highly mobile regions like the spinal cord, where fixation is required. The forces exerted by tethers during natural movements, combined with cable manipulation, can induce micro‐motions between the implant and surrounding tissue, compromising electrical interfaces and potentially causing tissue damage over time.

In contrast, untethered soft actuators eliminate the need for physical connections to external systems by utilizing wireless power transfer methods like inductive coupling, magnetic fields, or acoustic and light‐based stimulation. The absence of tethers offers advantages such as enhanced flexibility, reduced risk of complications, and improved patient comfort. Miniature untethered robots have shown significant promise in applications like on‐demand drug delivery and minimally invasive surgery. Their small size, active mobility, and remote controllability allow them to access hard‐to‐reach lesion sites, offering substantial potential for various biomedical applications. Advances in wireless, battery‐free technologies for neuroengineering, as highlighted in recent reviews,^[^ ^241^ ^]^ suggest that combining these fields could lead to fully implantable, reconfigurable bioelectronic devices capable of minimally invasive neural interfacing.

Despite these developments, untethered robotics face substantial limitations. One major difficulty is achieving effective and selective actuation within confined spaces like deep tissue environments, where different segments of the robot may need to perform distinct functions. Selective actuation is challenging without physical wires or channels. Some progress has been made by integrating materials that respond to specific stimuli, such as different wavelengths of light or varying rotational directions of magnetic fields. However, these approaches often add complexity to the material and structural design of the actuators. Focused ultrasound and NIR light have emerged as promising alternatives due to their deep tissue penetration capabilities (up to tens of centimeters for ultrasound and a few centimeters for NIR light) and millimeter‐level spatial resolution, making them viable options for selective actuation.^[^ ^76^ ^]^ Nonetheless, parameters like frequency/wavelength, intensity, and exposure duration must be carefully controlled to minimize risks of tissue heating or potential damage.

Another significant limitation of magnetic actuation is its reliance on bulky external equipment. Effective magnetic actuation typically requires large, powerful magnets or electromagnetic coils positioned outside the body. This dependence on external hardware not only limits the mobility and convenience of the actuation system but also restricts its use in medical settings, unencumbered access to the patient is important. Further, generating sufficient force from untethered soft robots remains a challenge. Most untethered miniature robots produce minimal force due to the limitations of wireless power sources and the high energy dissipation of soft materials. Given the force requirements for many biomedical applications, there is growing interest in wireless fluidic actuators and robots based on phase transitions, which have the potential to deliver considerably higher force outputs. These technologies may overcome current limitations, enabling more practical and effective biomedical interventions.

## 5 Conclusion and Discussion

The integration of responsive materials and soft actuators with bioelectronic devices offers a promising path toward minimally invasive, adaptable, and multifunctional biomedical implants. These innovations address significant challenges in interfacing with delicate biological tissues, offering improved biocompatibility, precise control, and the potential for enhanced therapeutic outcomes. In this Review, we presented several promising responsive materials and actuating mechanisms, each with distinct characteristics. We also highlighted examples that demonstrate the potential of shape‐morphing implants enabled by responsive materials and actuators for minimally invasive neural interfaces. While the field is still emerging, collaborative efforts across disciplines are accelerating progress. Key challenges remain in optimizing actuation mechanisms for in vivo environments, ensuring precise control, scaling down devices for implantation, and integrating multiple functionalities without compromising device performance.

Although many soft robotic bioelectronics succeed in proof‐of‐concept settings, clinical adoption requires a system‐level approach for true translational feasibility.^[^ ^24^, ^242^ ^]^ Critical considerations include power supply, control, form factor, safety, physical connections, durability under repeated use, and ease of integration. For instance, dielectric elastomer actuators operate at high voltages, whereas shape‐memory alloys need relatively high current; although both can be powered externally in the lab, long‐term implantation demands connectors or cables that risk infection. Compact batteries or wireless power transfer could mitigate this issue, while necessitating specialized circuitry, insulation, and rigorous biocompatible encapsulation to protect tissues and electronics from mutual damage. Similarly, magnetic actuation is untethered but may interfere with implanted devices like pacemakers or with imaging technologies such as MRI and still relies on external coils. Fluidic actuation produces large forces yet requires complex tubing and leak prevention strategies.

Long‐term operation within the body also calls for actuators that withstand mechanical fatigue, maintain stable output, and avoid catastrophic failure. Therefore, accelerated aging tests, cyclic loading evaluations, and real‐time monitoring are essential. Systems that demand complex or delicate surgical procedures may be impractical for routine clinical use. It is therefore critical to simplify the design and minimize the number of external leads. Different clinical targets impose varying requirements on actuators. For instance, vascular procedures might require sub‐millimeter and highly flexible devices for fluid environments, while brain implants may emphasize higher force output combined with soft, gentle structures.

While implantation often receives the most attention in medical device development, the extraction of these devices once their function is complete presents equally important challenges. Traditional implants, such as pacemakers and their leads, have well‐established surgical protocols, robust lead designs, and designated extraction tools. In contrast, soft implants or untethered micro‐robots could be more difficult to locate, grasp, or maneuver once implanted, because their deformable nature often requires more delicate handling. Dong et al. raised the significance of the explanting nerve cuff electrodes after use and proposed loosening thin film‐based nerve interfaces via electrochemical actuation strategy, thereby reducing nerve injuries, which is particularly useful for intraoperative nerve monitoring.^[^ ^71^ ^]^ Despite this, device explantation remains relatively underexplored compared to implantation. Going forward, we expect future research to focus on developing soft systems that can be tracked, navigated, and extracted without causing severe tissue injury or requiring invasive surgical procedures, thereby closing a critical gap in the clinical translation of soft robotic bioelectronic devices. Potential solutions include bioresorbable materials that gradually dissolve once the device's task is fulfilled, and integrated markers that facilitate visualization and retrieval.^[^ ^243^, ^244^ ^]^

Many of these soft actuation mechanisms remain at relatively low TRLs. For instance, pneumatic‐actuated bioelectronic devices have advanced to in vivo demonstrations in animal models (TRL 5–6), while magnetically actuated electronics largely remain at proof‐of‐concept stages (TRL 2–3). Further animal studies and eventual human trials will be required to ensure long‐term biocompatibility, robustness, and safety. Addressing these challenges calls for cross‐disciplinary efforts spanning materials science, electrical and mechanical engineering, bioengineering, and clinical medicine. Equally important is the collaboration between industry, academia, and regulators to transition lab‐scale soft actuators into safe, effective, and clinically viable solutions for bioelectronic medicine. Overcoming these barriers will help unlock the full potential of intelligent bioelectronic devices, revolutionize medical treatments, provide patient‐specific solutions and open new avenues in diagnostics and therapeutics.

## Conflict of Interest

The authors declare no conflict of interest.
